# A retrospective analysis of eye conditions among children attending St. John Eye Hospital, Hebron, Palestine

**DOI:** 10.1186/s13104-016-2011-9

**Published:** 2016-04-05

**Authors:** Riyad G. Banayot

**Affiliations:** St. John Eye Hospital, Jerusalem, Occupied Palestinian Territories

**Keywords:** Refractive errors, Conjunctivitis, Strabismus, Palestine

## Abstract

**Background:**

Eye diseases are important causes of medical consultations, with the spectrum varying in different regions. This hospital-based descriptive study aimed to determine the profile of childhood eye conditions at St. John tertiary Eye hospital serving in Hebron, Palestine.

**Methods:**

Files of all new patients less than 16 years old who presented to St. John Eye Hospital—Hebron, Palestine between January 2013 and December 2013 were retrospectively reviewed. Age at presentation, sex, and clinical diagnosis were extracted from medical records. Data were stored and analyzed using Wizard data analysis version 1.6.0 by Evan Miller. The Chi square test was used to compare variables and a *p* value of less than 0.05 was considered statistically significant.

**Results:**

We evaluated the records of 1102 patients, with a female: male ratio of 1:1.1. Patients aged 0–5 years old were the largest group (40.2 %). Refractive errors were the most common ocular disorders seen (31.6 %), followed by conjunctival diseases (23.7 %) and strabismus and amblyopia (13.8 %). Refractive errors were recorded more frequently and statistically significant (*p* < 0.001) among (11–15) age group. Within the conjunctival diseases category, conjunctivitis and dry eyes was more prominent and statistically significant (*p* < 0.001) among the 6–10 year old age group. Within the strabismus and amblyopia category, convergent strabismus was more common and statistically significant among the youngest age group (0–5 years old).

**Conclusions:**

The most common causes of ocular morbidity are largely treatable or preventable. These results suggest the need for awareness campaigns and early intervention programs.

**Electronic supplementary material:**

The online version of this article (doi:10.1186/s13104-016-2011-9) contains supplementary material, which is available to authorized users.

## Background

Visual impairment is a serious disability in children and its management is a priority of the World Health Organization’s VISION 2020 campaign: The Right to Sight [1]. Several ocular morbidity studies have estimated the magnitude of eye diseases among children. In the United States of America, the most common vision disorders reported among children were strabismus, amblyopia and optical problems [2]. In Nigeria, refractive errors, vernal conjunctivitis, eye injuries, and corneal inflammation were the leading causes of childhood eye morbidity [3]. In a study in Tikrit, Iraq, allergic conjunctivitis, refractive errors, ocular trauma, infection, squint and nasolacrimal duct obstruction were the most common ocular conditions treated in an outpatient department [4].

Data on patterns of eye diseases in children might be useful in improving any existing primary eye care facilities and provides useful information for planning child eye care services in a given region or the whole of a country. Understanding the specific causes of visual reduction also helps in proper and efficient allocation of resources for preventive and control measures as well as treatment.

Therefore, this hospital-based descriptive study, besides being the first of its kind in the region, aimed to determine the distribution and spectrum of childhood eye diseases at St. John Eye tertiary hospital serving in Hebron, Palestine between January 2013 and December 2013.

## Methods

Patient archived data of all new patients less than 16 years old who presented to St. John Eye Hospital—Hebron, Palestine between January 2013 and December 2013 were electronically produced and retrospectively reviewed. St. John Eye Hospital—Hebron serves as a referral center for the Hebron Governorate (size: 1060 km^2^ with a population of approximately 70,000 inhabitants). In Hebron, primary care is provided by Ministry of Health clinics and hospitals. St. John also provides primary and secondary eye care services to walk-in patients. At the first visit, all patients had a full ophthalmic evaluation, including refraction/cyclorefraction, carried by and optometrist and an assessment of ocular motility, intraocular pressure (IOP), slit lamp examination and dilated ophthalmoscopy carried out by a consultant ophthalmologist. Tests were conducted to elicit a diagnosis, and management initiated as required. Consultations to subspecialists were made when necessary. At the end of the consultation, one or more diagnoses are recorded in each patient’s file.

Age at presentation, sex, and clinical diagnosis were extracted from medical records. One main diagnosis is reported for each patient in the study, which represents the diagnosis that is primarily responsible for the outpatient services provided. The clinical diagnoses were grouped as appropriate into anatomical categories; lid diseases, lacrimal diseases, orbital diseases, conjunctiva, cornea/sclera, lens, glaucoma, iris/ciliary body/choroid, retina/vitreous, diabetes mellitus, neuro-ophthalmic diseases, strabismus and amblyopia, congenital disorders, refractive error, ocular trauma, normal exam and miscellaneous. Additional file 1: Table S1 shows the categories and subcategories used. Patients were grouped by age into preschool (0–5 years), school-age (6–10 years) or older children (11–15 years) groups. Patients who presented for a medical check-up and had no eye disorders were excluded from the study. Data were stored and analyzed using Wizard data analysis version 1.6.0 by Evan Miller. The Chi square test was used to compare variables and a *p* value less than 0.05 was considered statistically significant. Ratios and percentages were calculated and tabulated. Possible associations with age and gender were pursued using p values. The results were described, summarized and presented in tables. These were followed by interpretations of the results, discussions and conclusions.

Ethical approval and permission to conduct the study was obtained from St. John Eye hospital Ethics committee. Written informed consent was not obtained since confidentiality of the study was maintained by concealing the names of patients on archived data sheets.

## Results

**Fig. 1:**
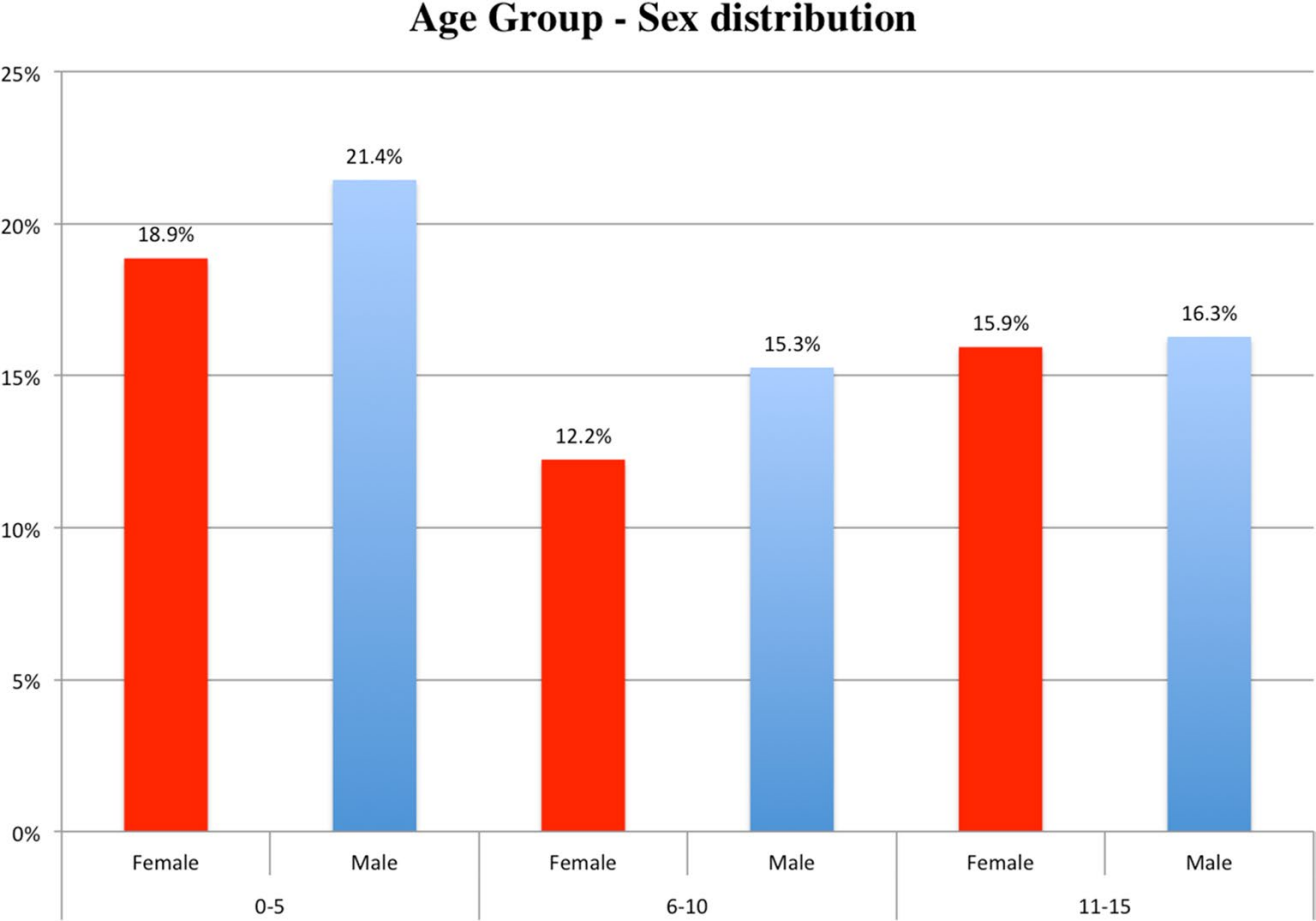
Age group and sex distribution. Female and Male percentages across age groups

We evaluated 1102 records of patients who were seen in the hospital during the study period (January 2013–December 2013), constituting 100 % of all new patients seen less than 16 years old. Patients who had no eye disorders (215 patients) represented 19.5 % of all patients and were excluded from the study. The review was done for 887 patients with a mean age of 7.14 years. There were 417 females (47 %) and 470 males (53 %), resulting in a female: male ratio of 1:1.1. The highest frequency of consultation was recorded among the (0–5) age group and constituted 40.2 % of patients. Figure 1 shows age group and sex distribution with male predominance in all three age groups.

**Fig. 2:**
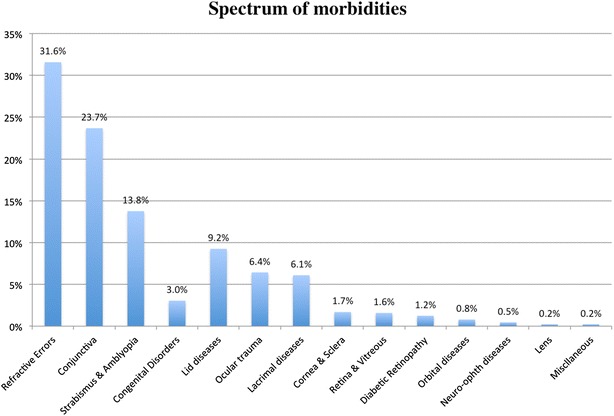
Spectrum of morbidities. Percentages of morbidities in study

Refractive errors were the most common disorders seen (31.6 %), followed by conjunctival diseases (23.7 %) and strabismus and amblyopia (13.8 %). Figure 2 shows spectrum and percentages of morbidities seen.

The frequency and pattern of eye diseases varied across age groups. The difference in presentation by age group was more common among 0–5 year-old children compared to other age groups (Chi square, *p* < 0.001) for convergent strabismus and nasolacrimal duct obstruction. Dry eyes were more common among 6–10 year-old children compared to other age groups (Chi square, *p* < 0.001). Refractive errors and blepharitis were more common among 11–15 year-old children compared to other age groups (Chi square, *p* < 0.001). Additional file 2: Table S2 shows distribution of eye morbidity across age groups.

Refractive errors were the most common disorders seen representing 31.6 % of cases. Refractive errors were recorded more frequently among the (11–15) age group compared to other age groups (Chi square, *p* < 0.001).

Conjunctival diseases were the second most common disorder seen and represented 23.7 % of cases. Within this category, dry eye (Sicca) was more prominent among the (6–10) age group compared to other age groups (Chi square, *p* < 0.001).

Strabismus and amblyopia was the third most common disorder seen and represented 13.8 % of cases. Convergent strabismus was more common among the (0–5) age group compared to other age groups (Chi square, *p* < 0.001).

Lid diseases were the fourth most common presentation in our study representing 9.2 % of cases. Blepharitis was more common among the (11–15) age group compared to other age groups (Chi square, *p* < 0.001).

Ocular trauma was the fifth most common presentation in this study and represented 6.4 % of cases.

Lacrimal diseases were sixth most common presentation in this study and represented 6.1 % of cases. Naso-lacrimal duct obstruction was more prominent among the (0–5) age group (Chi square, *p* < 0.001).

Cornea and scleral diseases represented 1.7 % of cases (n = 15), with keratoconus (n = 10) as the most common condition in this category. Retina and vitreous disorders represented 1.6 % (n = 14) of cases. Diabetic patients represented 1.2 % (n = 11) of cases and the majority (n = 8) showed no retinopathy. Orbital diseases represented 0.8 % of cases (n = 7). Neuro-ophthalmic diseases represented 0.5 % of cases (n = 4). Lens disorders and miscellaneous disorders each represented 0.2 % of cases (n = 2).

Conditions, which did not fit in any specific subcategory, were labeled as “other”. Additional file 3: Table S3 shows these conditions among age groups and their numbers.

This study showed that there were no statistically significant differences in prominent conditions in either females or males (Chi square, *p* = 0.064). Additional file 4: Table S4 shows distribution of conditions according to sex and age group.

## Discussion

To our knowledge, this is the first study on the spectrum of childhood eye diseases in a sample from a referral center in Palestine. Convenience sampling (proximity and availability) prevents this study from assuming to be a representative of the population.

Refractive errors were the most common disorders seen in this study (31.6 %) and were the most common morbidity seen among the (11–15) age group compared to other age groups (Chi square, *p* < 0.001). These results are consistent with reports from Nigeria [3], China [5], Bengal [2], Kathmandu [6] and India [7]. Refractive errors affect childhood development, and without preschool or school eye screening for refractive errors, many children with refractive errors go unnoticed.

Conjunctival diseases (conjunctivitis representing 68 % of this category) were the second most common morbidity seen for all age groups in this study (23.7 %). Previous reports of allergic conjunctivitis as the most common disorder in children [4, 8–10] and the second most common in children [3, 5, 11–13] are supported by this study.

Strabismus and amblyopia was the third most common morbidity seen in this study representing 13.8 % of cases. Strabismus (convergent, divergent and vertical) represented 96 % of cases in this category. Convergent strabismus was more common among the (0–5) age group compared to other age groups (Chi square, *p* < 0.001). Reports from Katmandu [6] and India [7] show strabismus as the second most common presentation but with much lower frequencies 1.6 and 2.5 % respectively. A report from Iraq [4] showed strabismus as the fifth most common presentation and represented 12.1 % of cases.

Lid diseases were the fourth most common presentation in our study representing 9.2 % of cases. Blepharitis was more common among the (11–15) age group compared to other age groups (Chi square, *p* < 0.001).

Ocular trauma was the fifth most common presentation in this study and represented 6.4 % of cases with the majority of trauma cases (70 %) being minor injuries (e.g., unintentional blows to eye, foreign bodies getting in the eye). The female to male ratio for eye injuries was 1:1.9. Our results are different from other reports where ocular trauma in children was the first in Nigeria [11] and second in Ethiopia [10]. These differences could be due to different study designs, populations studied etc. Most studies [3, 4, 8, 9, 12] reported trauma as the third most common presentation. Eye injuries remain a major cause of unilateral visual impairment worldwide and a common cause of non- congenital unilateral blindness [14]. Children are particularly at risk of ocular injury due to their decreased ability to detect and avoid potential hazards [15]. Most childhood eye injuries are sustained during unsupervised play and domestic activities. The challenge of managing childhood eye injuries is enormous with considerations ranging from late presentation to eye care centers to a lack of facilities, the low socioeconomic status of the children involved, the special care required during examination, postoperative management and the risk of secondary vision loss [8, 15]. Prevention of ocular trauma in children remains a priority in order to reduce ocular morbidity [15]. This will involve the adequate education of children, parents and teachers to ensure adequate supervision during playtime.

Lacrimal diseases were sixth most common presentation in this study and represented 6.1 % of cases. Naso-lacrimal duct obstruction (NLDO) was more prominent among the (0–5) age group compared to other age groups (Chi square, *p* < 0.001). This finding is supported by similar findings from Iraq [4].

Limitations of our study include a retrospective study design, hospital-based, and selection bias. These limitations could have increased the number of cases and, consequently, overestimated the incidence of eye diseases in our sample compared to the population. However, the strength of our study include that it is the first study at SJEH-Hebron giving a general view of ocular findings from a specialty eye clinic.

## Conclusions

Across the study period, the most common causes of ocular morbidity in decreasing frequency were refractive errors, conjunctivitis, strabismus, blepharitis, ocular trauma and NLDO. These morbidities were preventable (e.g., trauma) through proper eye health education to the community or treatable medically and/or surgically to prevent visual impairment and blindness. Programs that raise the capacity of health providers and screening programs should be conducted regularly and preferably conducted in partnership with all health service providers. It is also of equal importance the early presentation of these children to eye care centers for management. For this, teachers should be oriented and trained in identifying common eye problems among school children so that these children can be referred for prompt treatment.

## Additional files

10.1186/s13104-016-2011-9 Categories and subcategories used in study.
10.1186/s13104-016-2011-9 Morbidities, diagnosis and age groups. Most common morbidities including sub-categories are shown. Categories excluded: cornea and sclera, diabetic retinopathy, dislocated lens, neuro-ophthalmic diseases, orbit, retina and vitreous, miscellaneous causes.
10.1186/s13104-016-2011-9 “Other” conditions.
10.1186/s13104-016-2011-9 Conditions according to sex.
